# Evaluation of SARS-CoV-2 interferon gamma release assay in BNT162b2 vaccinated healthcare workers

**DOI:** 10.1371/journal.pone.0303244

**Published:** 2024-05-10

**Authors:** Angélica Ramos, Sandra Martins, Ana Sofia Marinho, Pedro Norton, Maria João Cardoso, João Tiago Guimarães

**Affiliations:** 1 Serviço de Patologia Clínica, Centro Hospitalar Universitário de São João, Porto, Portugal; 2 EPIUnit–Instituto de Saúde Pública, Universidade do Porto, Porto, Portugal; 3 Laboratório para a Investigação Integrativa e Translacional em Saúde Populacional (ITR), Universidade do Porto, Porto, Portugal; 4 Serviço de Saúde Ocupacional, Centro Hospitalar Universitário de São João, Porto, Portugal; 5 Departamento de Biomedicina, Faculdade de Medicina, Universidade do Porto, Porto, Portugal; Korea Disease Control and Prevention Agency, REPUBLIC OF KOREA

## Abstract

To predict protective immunity to SARS-CoV-2, cellular immunity seems to be more sensitive than humoral immunity. Through an Interferon-Gamma (IFN-γ) Release Assay (IGRA), we show that, despite a marked decrease in total antibodies, 94.3% of 123 healthcare workers have a positive cellular response 6 months after inoculation with the 2nd dose of BNT162b2 vaccine. Despite the qualitative relationship found, we did not observe a quantitative correlation between IFN-γ and IgG levels against SARS-CoV-2. Using stimulated whole blood from a subset of participants, we confirmed the specific T-cell response to SARS-CoV-2 by dosing elevated levels of the IL-6, IL-10 and TNF-α. Through a 20-month follow-up, we found that none of the infected participants had severe COVID-19 and that the first positive cases were only 12 months after the 2nd dose inoculation. Future studies are needed to understand if IGRA-SARS-CoV-2 can be a powerful diagnostic tool to predict future COVID-19 severe disease, guiding vaccination policies.

## Introduction

Serological assays that evaluate antibody responses are, so far, more used to monitor the immune response to SARS-CoV-2, than cellular assays that measure T-cell responses [1]. This can be explained because there are many more commercial serological tests available than cellular ones, they are less expensive, easy to perform and, mainly, can be performed on stored serum, whereas cellular tests require fresh whole blood.

However, some studies have shown that T-cell responses are more sensitive markers of past SARS-CoV-2 infection compared with antibody responses and that have a greater correlation with the protective immunity [2–7]. Although some studies have shown the usefulness of serological tests to monitor the immune response to SARS-CoV-2, others conclude that they may not accurately predict the immunity to SARS-CoV-2 in some subgroups of the population, for example in immunocompromised patients with impaired B-cell function [2, 8–12]. Additionally, T-cell responses were shown to be more robust than antibody responses in convalescents with mild or asymptomatic COVID-19 infection [6, 7, 13, 14]. Thus, clinical T-cell assays are needed to evaluate the cell-mediated immune response against SARS-CoV-2 as evidence for past infection and immune response to vaccination [14].

Interferon gamma (IFN-γ) release assay (IGRA) is blood diagnostic test used to measure IFN-γ released by T-cells after stimulation with pathogen-specific antigens. IGRA is best known for its role in diagnosing latent *Mycobacterium tuberculosis* infection [15, 16]. The IGRA-TB was the first T-cell–based immunodiagnostic test used in clinical practice in the field of infectious diseases [17]. More recently the IGRA-CMV has shown promising results for risk stratification of CMV infection or diagnosis in immunocompromised patients [18–23].

Early in March 2020, several serological tests for SARS-CoV-2 were already available, in several formats and methodologies (ELISA, CLIA, lateral flow test), detecting IgA, IgM, IgG or Total Ig through several immunogenic target proteins. However, only at the beginning of 2021 the first tests of IGRA-SARS-CoV-2 emerged.

Here, we evaluated the cellular response of a set of healthcare workers, 6 months after the 2nd dose of BNT162b2 vaccination through the *SARS-CoV-2 Interferon-Gamma Release Assay (IGRA)* (EUROIMMUN, Lübeck, Germany). Furthermore, we correlated the T-cell response to the antibodies response, namely those against the S1 protein of SARS-CoV-2.

## Material and methods

### Study design

Through a set of 123 healthcare workers from Centro Hospitalar Universitário de São João (CHUSJ), characterized in Table 1 (S1 File), we evaluated humoral immunity to SARS-CoV-2 at the first and sixth month after the inoculation of two BNT162b2 vaccine doses. At the sixth month, we also assessed cellular immunity.

**Table 1:** Study population characterization.

**A.**						
	Healthcare Workers (n = 123)					
	**n**			**%**		
**Smokers**	19			15.4		
**Comorbidities**						
Hypertension	18			14.7		
Asthma	8			6.5		
Autoimmune disease	12			9.8		
Allergies	5			4.0		
Diabetes mellitus	3			2.4		
Cancer	3			2.4		
Cardiovascular disease	4			3.3		
Primary biliary cirrhosis	1			0.8		
Hereditary anaemia	1			0.8		
Arthrosis	1			0.8		
Ulcerative colitis	1			0.8		
Idiopathic thrombocytopenia	1			0.8		
Dyslipidaemia	1			0.8		
**B.**						
	Healthcare Workers (n = 84)					
**Hemogram**	Median	Min–Max	Mean	SD	Reference value	Unit
RBC	4.5	3.6–5.4	---	---	4.0–5.0	x 10^12^ /L
Hemoglobin	13.5	11.5–15.3	---	---	12.0–16.0	g/dL
Globular volume	41.0	34.5–45.7	---	---	37–49	%
MCV	90.5	78.1–102.2	---	---	87–103	fL
MCH	30.4	25.6–32.4	---	---	27–35	pg
MCHC	33.3	30.9–35.4	---	---	28–36	g/dL
RDWCV	12.8	11.7–15.2	---	---	11–16	%
RDWSD	42.3	38.5–47.6	---	---	37–54	fL
Leucocytes	---	---	6.7	2.4	4.0–11.0	x 10^9^ /L
% Neutrophils	---	---	62.9	59.9	53.8–69.8	%
% Eosinophils	2.4	0.0–10.2	---	---	0.6–4.6	%
% Basophils	---	---	0.5	0.4	0.0–1.5	%
% Lymphocytes	34.4	4.3–67.3	---	---	22.6–36.6	%
% Monocytes	6.8	2.1–11.6	---	---	4.7–8.7	%
% Immature granulocytes	---	---	0.2	0.1	< 0.5	%
Platelet count	247	171–373	---	---	150–400	x 10^9^ /L
Platelet volume	0.26	0.18–0.39	---	---	---	%
MPV	10.5	8.4–12.7	---	---	---	fL
PDW	12.4	9.1–16.8	---	---	---	fL
PLCR	29.1	11.8–46.8	---	---	---	%
**Hepatic biomarkers**						
AST	20	14–78	---	---	10–31	U/L
ALT	16	9–65	---	---	10–31	U/L
**Renal biomarkers**						
Urea	---	---	34.4	8.4	10–50	mg/dL
Creatinine	0.67	0.48–139	---	---	0.51–0.95	mg/dL

Table 1 shows the characterisation of the population based on comorbidities (1A) and analytical parameters (1B). Table 1A shows the comorbidities reported by all the participants. Table 1B shows the analytical parameters of the 84 participants who, in addition to determining the immune response to SARS-CoV-2, also voluntarily measured liver and kidney biomarkers and performed a hemogram. The mean or median of all the analytical parameters accessed is within the reference values, suggesting a healthy population. RBC–Red Blood Cells; MCV–Mean Corpuscular Volume; MCH–Mean Corpuscular Hemoglobin; MCHC–Mean Corpuscular Hemoglobin Concentration; RCDWCV–Red Cell Distribution Width based on Coefficient of Variation; RCDWSD–Red Cell Distribution Width based on Standard Deviation; MPV–Mean Platelet Volume; PDW–Platelet Distribution Width; PLCR–Platelet-Large Cell Ratio; AST–Aspartate Aminotransferase; ALT–Alanine Aminotransferase.

The inoculation dates of the first, second and booster doses in CHUSJ were the following: December 2020, January 2021 and November 2021, respectively. The study population corresponds to 112 women and 11 men, with a mean age of 48 years old (range 22–67) and 48 years old (range 23–67), respectively. Access to humoral immunity was performed through an immunoassay for the determination of SARS-CoV-2 antibodies against the S1 protein. In addition, to exclude a SARS-CoV-2 infection, an immunoassay was performed for the determination of antibodies against N protein, as these are absent in the vaccine-induced immune response. Since the participants in this study belong to an even larger group of healthcare workers in which the vaccine response was monitored from the day after the 1st dose, we had the opportunity to measure anti-N antibodies at the following time points: 1 month after the 1st dose, 1 month after the 2nd dose, 3 months after the 2nd dose and 6 months after the 2nd dose. Access to cellular immunity was performed using a SARS-CoV-2 Interferon-Gamma Release Assay (IGRA). To ensure the T-cell response, IL-6, IL-10 and TNF-α levels were also measured in the stimulated T cell-specific plasma (via antigens based on the SARS-CoV-2 spike protein) from 18 participants. This subset corresponds to 17 females with a mean age of 50 years old (range 29–62) and 1 male with 58 years-old. These 18 participants were selected on the basis of their IgG levels at 6 months after the 2nd dose, corresponding to the 9 participants with the highest levels and the 9 participants with the lowest levels.

Through a 20-month follow-up, we also investigated who tested positive for COVID-19 after the 2nd dose. Additionally, we investigated the date of infection and the severity of the disease by the need for hospitalization.

All healthcare workers enrolled in the study completed an epidemiological survey in which they were asked about a known underlying disease or daily medication. All tests were performed according to the manufacturer’s instructions. A written informed consent was obtained from all subjects. The Ethics Committee of Centro Hospitalar Universitário de São João approved this study (CE 130–20, 30 April 2020).

### Assays

#### SARS-CoV-2 IgG II quant

The SARS-CoV-2 IgG assay (Abbott Laboratories, Illinois, USA) is a chemiluminescent microparticle immunoassay (CMIA) for the qualitative detection of IgG against SARS-CoV-2. The test can be performed in human serum and plasma and uses the RBD of the S1 subunit of the SARS-CoV-2 spike protein as the immunogenic protein. Results < 50.0 AU/mL are reported as negative and ≥ 50.0 AU/mL as positive. The measuring range for the assay is 21–40000 AU/mL.

#### BioPlex 2200 SARS-CoV-2 IgG panel

The BioPlex 2200 SARS-CoV-2 IgG Panel (BIO-RAD, Hercules, California, USA) is a multiplex immunoassay for the qualitative detection and semi-quantitative differentiation of IgG against the RBD, S1, S2 and N proteins of SARS-CoV-2. Results *<* 10U/mL are reported as negative and ≥ 10 U/mL as positive. The measuring range for the assay is 1–100 U/mL. Results outside of this range are reported as *<* 1 U/mL or *>* 100 U/mL.

#### Quan-T-Cell system

The Quan-T-Cell System combine the Quan-T-Cell ELISA and the Quan-T-Cell SARS-CoV-2 (Euroimmun, Lübeck, Germany) for the quantitative determination of IFN-γ released by T cells specific for SARS-CoV-2. Briefly, 0.5 mL of fresh heparinized whole blood was added to 3 test tubes: (1) CoV-2 IGRA BLANK: no T-cell stimulation, for assess of the IFN-γ background; (2) CoV-2 IGRA TUBE: specific T-cell stimulation by antigens of SARS-CoV-2 spike protein; (3) CoV-2 IGRA STIM: unspecific T-cell stimulate on by means of a mitogen, for control of the stimulation ability. After sampling, the test tubes were inverted six times and incubated for 16 to 20 hours at 37°C. The samples were then centrifuged at 12000 RCF for 10 minutes and the plasma supernatant was transferred into an eppendorf and stored at– 20°C until testing. IFN-γ was finally detected in the supernatants by an enzyme-linked immunosorbent assay (ELISA) using the Euroimmun Analyzer I instrument (Euroimmun, Lübeck, Germany). IFN-γ response was measured by subtracting the result in the stimulated tube minus the result in the unstimulated tube. The test validation criteria were as follows: IFN-γ [blank] < 400 mIU/mL and IFN-γ [stim]–IFN-γ [blank] > 400 mIU/mL. Results were interpreted as follows: IFN-γ [SARS-CoV-2]–IFN-γ [blank] < 100 mIU/mL negative, 100–200 mIU/mL borderline, and > 200 mIU/mL as positive.

#### Human High Sensitivity T Cell Magnetic Bead Panel

The Human High Sensitivity T Cell Magnetic Bead Panel Kit (# HSTCMAG– 285K, Milliplex MAP Kit, Millipore, Germany) associated with the Luminex 200TM xMAPTM Technology (Luminex Corp., Austin, USA) is a quantification methodology based on the Median Fluorescence Intensity (MFI) data using a standard five parameter logistic (5-PL) curve fit created by the Luminex xPONENT Software (version 3.1).

### Data analyses

For variables with a non-normal distribution, the Mann-Whitney U-test was applied. Correlation tests were carried out using Spearman’s Rho test. Multivariate adjusted linear regression analysis (Chi-Square) was carried out to correct the relationship between IGRA, age and gender. Differences between the samples were considered significant at p < 0.05.

## Results

**Fig 1:**
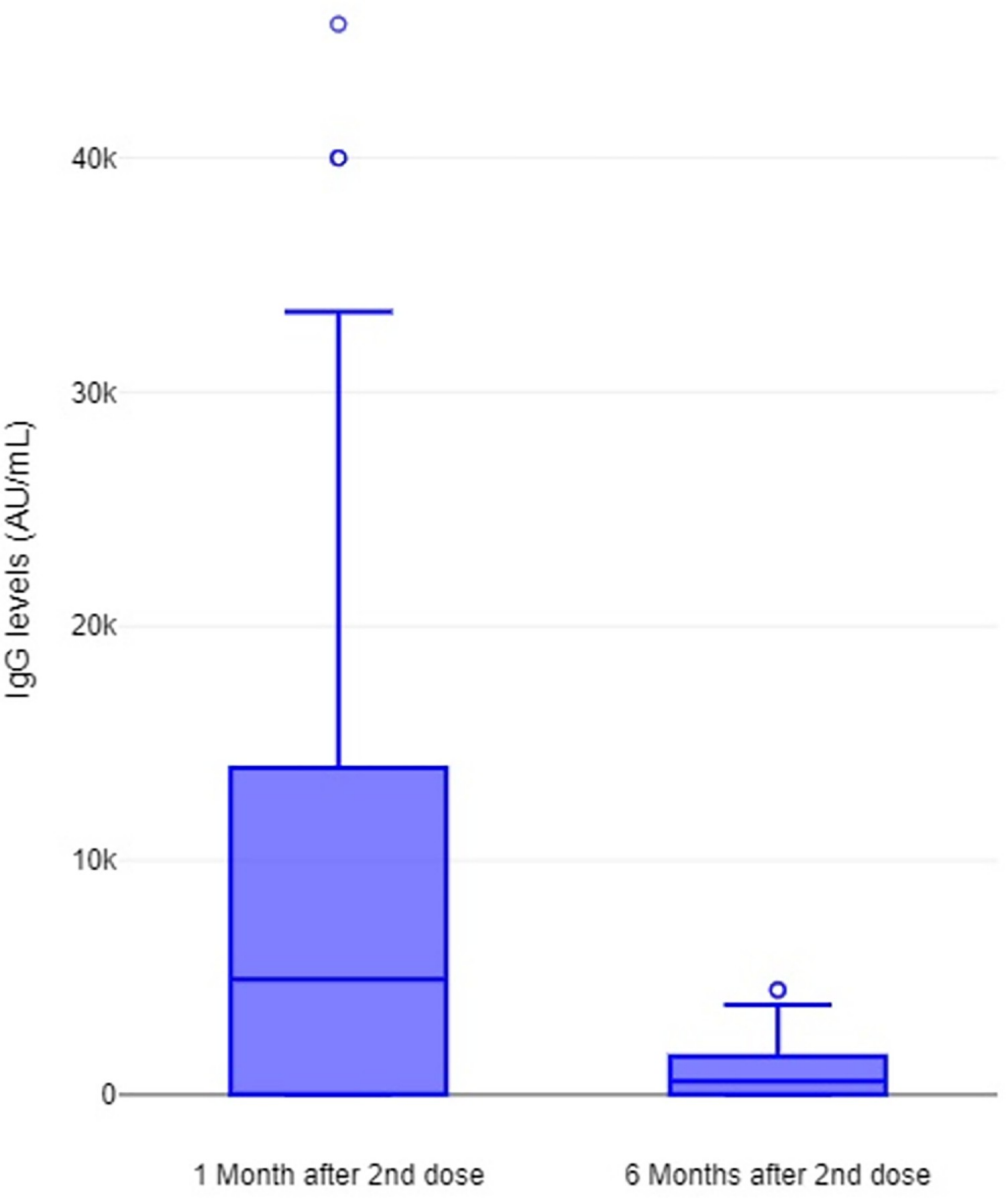
Evolution of anti-S1 IgG levels after vaccine inoculation. This graph shows the quantitative evolution of anti-S1 IgG over time. In it we can see that antibody levels decrease from an average of 15358 AU/mL (range 341–4502) to one of 1662 AU/mL (range 2113–45715), from the first to the sixth month, respectively, after the inoculation of the 2nd dose.

Six months after the second dose of the BNT162b2 vaccine, we found that all participants showed a positive humoral immune response to SARS-CoV-2 (anti-S1 IgG ≥ 50.0 AU/mL). The mean serological titer found was 1662 AU/mL (range 341–4502), which corresponds to 10% of the amount of antibodies dosed 1 month after the 2nd dose (Mean: 15358 AU/mL, range 2113–45715) (Fig 1). The production of SARS-CoV-2 IgG anti-N antibodies was not found in any of the participants, excluding the possibility of past, non-reported or asymptomatic SARS-CoV-2 infection (S2 File).

Regarding to cellular immune response, we found that 94.3% (116/123) had IFN-γ release values ​​> 200 mIU/mL and were considered positive according to Quan-T-Cell System protocol. Among the positives, the mean value was 1439 (IQR 77–2500) for IFN-γ production. Concerning the 7 non-positive results: 2 were negative (< 100 mIU/mL), 2 were borderline (100–200 mIU/mL) and 3 were invalidated due to a value of IFN-γ [blank] > 400 mIU/mL.

**Fig 2:**
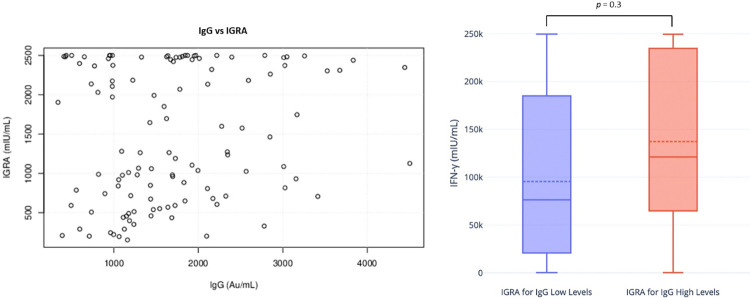
Correlation between IgG and IGRA levels, 6 months after vaccination. The scatter plot on the left shows the correlation between IgG and IGRA levels, 6 months after inoculation of the 2nd dose of the BNT162b2 vaccine. Although we found a quantitative correlation (94.3% agreement), there is no quantitative correlation between humoral and cellular immunity, according to Spearman’s correlation analyses (rho = 0.14). The Box–and–Whiskers plot on the right shows the relationship between low and high IgG levels and IGRA levels. Low and high levels were considered to be those below and above the IgG mean, respectively (IgG: 1662 AU/mL, mean). Although we found a tendency for higher IgG values to correspond to higher IGRA levels, this difference did not prove statistically significant (*p*-value *=* 0.3).

Although we found a qualitative relationship between IgG and IFN-γ results (94.3% of agreement), we did not find a quantitative relationship between both immunoassays (S3 File). Through Spearman correlation, we found a rho = 0.14, reflecting a non-association between IgG and IFN-γ levels (Fig 2). Indeed, when we compared IGRA values between participants with IgG values below and above the mean (1662 AU/mL), we did not find a statistically significant difference between the two groups (Fig 2).

We found a weak negative correlation between IGRA levels and age (rho = - 0.23; *p*-value = 0.000) and no statistically significant difference between IGRA levels from men and women (*p*-value = 0.202) (S6 File). The multivariable-adjusted linear regression analysis (Chi Square) that was also performed confirms these results (IGRA-age, *p*-value = 0.002; IGRA-sex, *p*-value = 0.133).

**Fig 3:**
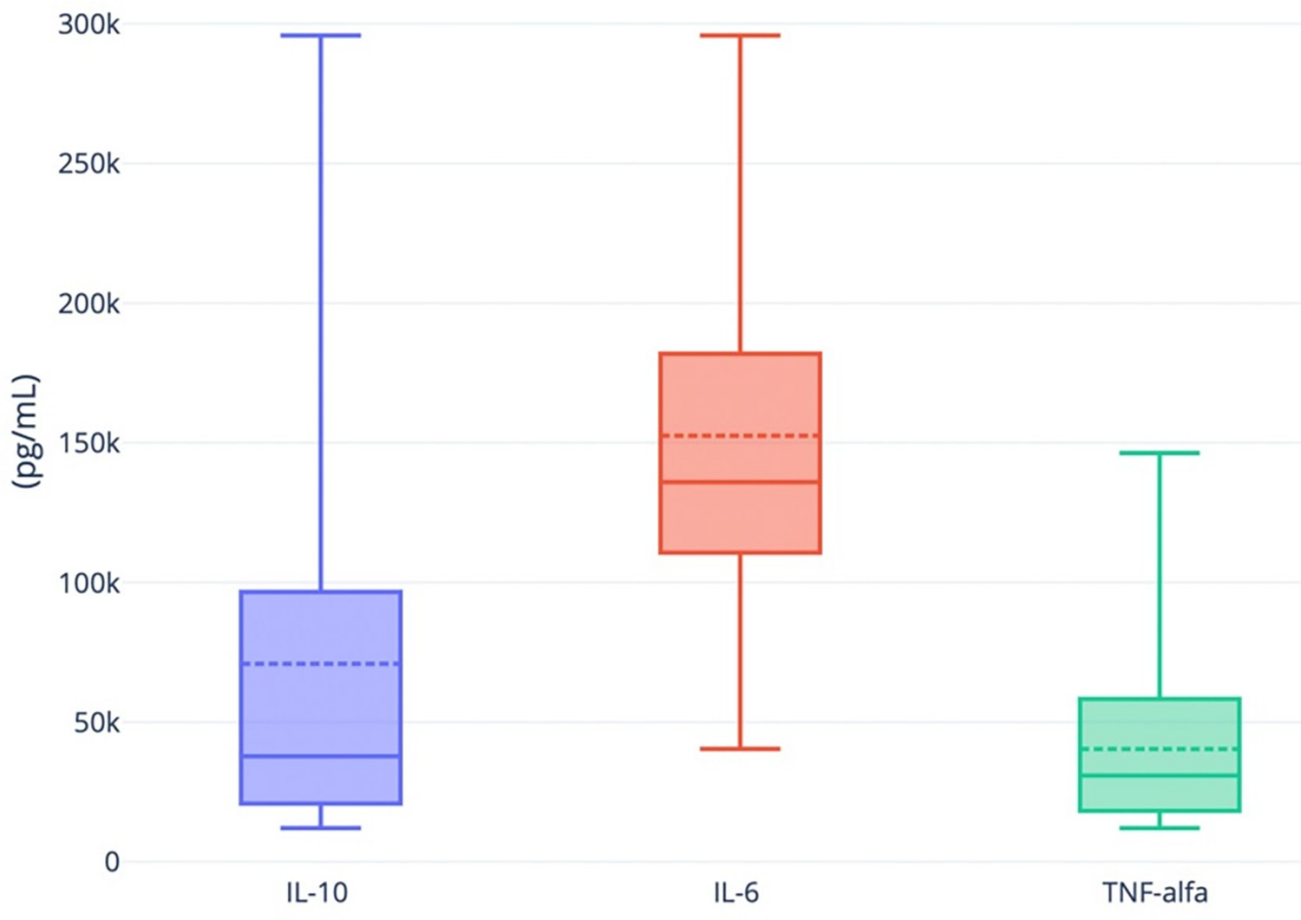
The interleukins levels after 6 months after vaccination. The box plot shows the levels of IL-6, IL-10 and TNF-α assessed from a subgroup of 18 healthcare workers. These interleukins were measured from T-cell-specific stimulated plasma (via antigens based on the SARS-CoV-2 spike protein) from CoV-2 IGRA TUBE. Although there was no control group, all molecules were produced at very high levels, confirming a strong T-cell response 6 months after BNT162b2’s two-dose vaccine regimen was complete.

Regarding, IL-6, IL-10 and TNF-α, we found positive levels of these molecules in the 18 participants studied (Fig 3 and S4 File). The highest values were found for IL-6 (median = 915.8 pg/mL), followed by TNF-α (median = 200.9 pg/mL) and, finally, IL-10 (median = 70.9 pg/mL). For this subgroup, the average values found for IgG and IGRA were 1798.6 AU/mL and 1242.5 mIU/mL, respectively. No correlation was found between these proteins (IGRA-IL-6: rho = 0.13, *p* = 0.60; IGRA-IL-10: rho = 0.02, *p* = 0.95; IGRA- TNF-α: rho = - 0.11, *p* = 0.65; IgG-IL-6: rho = - 0.04, *p* = 0.87; IgG-IL-10: rho = - 0.04, *p* = 0.87; IgG- TNF-α: rho = 0.19, *p* = 0.40).

**Fig 4:**
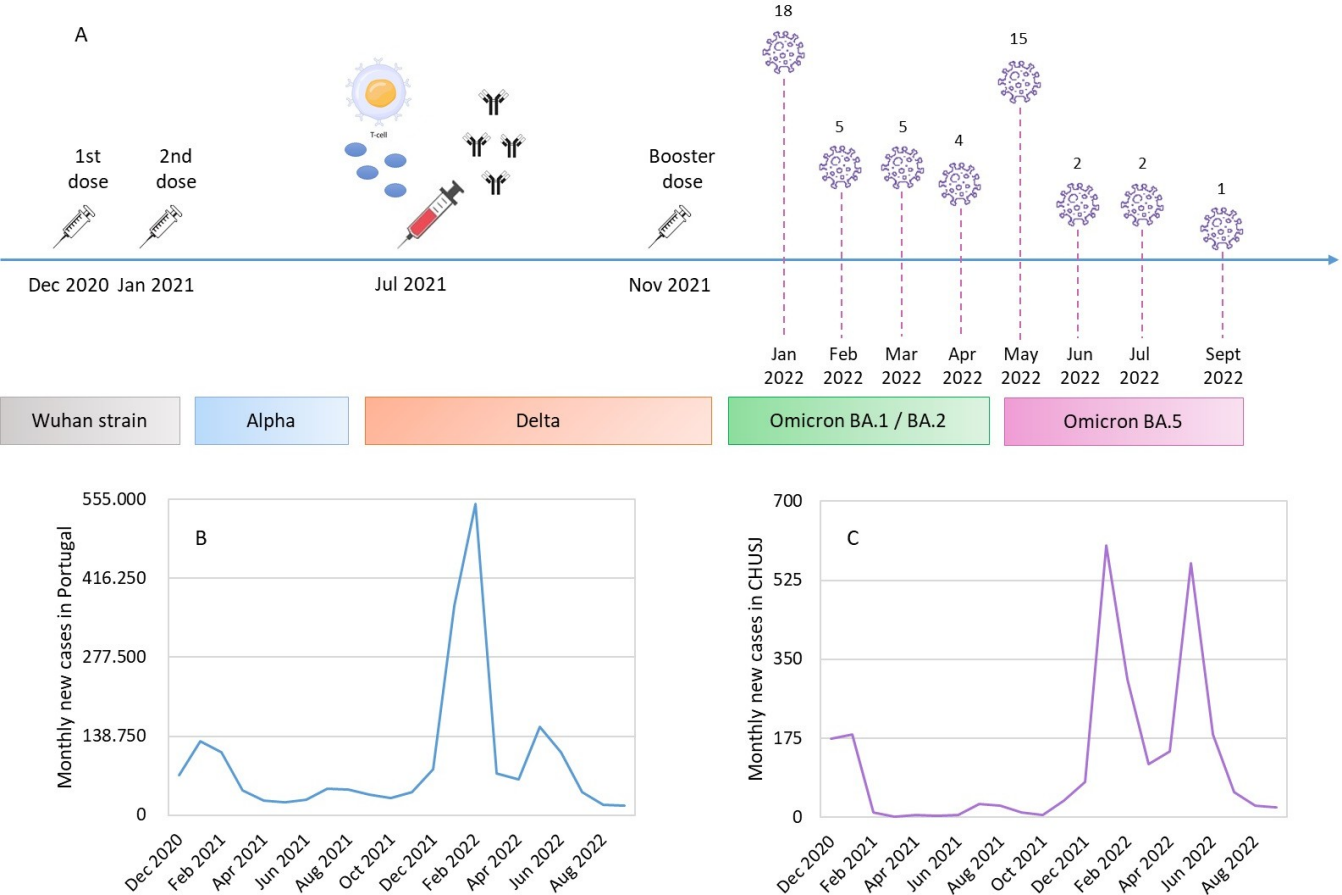
Healthcare workers follow-up, 6 months after vaccination. The scheme shows the study timeline (A). The 1st dose was administered on 27 December 2020 and the 2nd dose on 27 January 2021. The samples for IgG and IGRA analysis were collected at the end of July 2021. Through a follow-up of 20 months, we found that the first infections in the enrolled professionals were only one year after the 2nd shot. The months with most cases were January and May 2022. Interestingly, in graphs B and C, we can see that these two periods corresponded to infection peaks both at national level (B) and in healthcare workers from CHUSJ in general (C). Graph B shows the average number of new cases per month and Graph C the total number of new cases per month. The swift of variants in this period may explain these pronounced increases. In particular, as we can see in the scheme, in January 2022 the Omicron BA.1/BA.2 variant becomes dominant over the Delta, and in May 2022 the Omicron BA.5 strain becomes the most frequent in Portugal, replacing the BA.1/BA.2 variant. Graph B is based on data from the Directorate-General for Health (Portugal). Data on the prevalence of the different variants in Portugal were based on epidemiological reports from the National Institute of Health Doctor Ricardo Jorge.

Through a 20-month follow-up, we found that 43% (53/123) of participants had COVID-19 but none of them had a severe illness that required hospitalization. The first infected participants were positive at 12 months after the 2nd dose. Most infections occurred at 12 (n = 18) and 16 (n = 15) months later, in January and May 2022, respectively (S5 File). The difference between the IGRA levels of those with and without infection was not statistically significant (*p*-value = 0.5). The results are shown in Fig 4.

## Discussion

Our study shows that 94.3% of the healthcare workers enrolled keep a positive cellular immune response to SARS-CoV-2, despite a decrease of 90% in antibodies, 6 months after the 2nd dose of BNT162b2 vaccine (Fig 1). These findings are in line with those described by other authors [24–27]. Namely, in a study published in Science on December of 2021, Goel and collegues reported that despite antibodies declined from peak levels, they remain detectable in most subjects at 6 months after mRNA vaccination and that vaccine-induced antigen-specific CD4+ and CD8+ T-cells persists over the same period of time [27]. These 6 months of persistent cellular response after COVID-19 vaccination was also described by Guerrera *et al* and a set of previous studies that investigated the cellular immunity after SARS-CoV-2 infection [14, 26, 28, 29].

After asking participants through an epidemiological survey, we did not find a relationship between the 7 non-positive results and an underlying pathology or therapy. For IGRA-TB, it is known that medical treatments or conditions that impair immune functionality and other immunological variables can potentially reduce IFN-γ responses [30–32]. For example, an insufficient amount of lymphocytes, a presence of autoantibodies to IFN-γ or of heterophile antibodies, and non-specific secretion of IFN-γ may be associated with a low mitogenic response or a high response in the blank tube [33, 34].

We also assessed a possible correlation between cellular and humoral responses (Fig 2). In line with other studies, although we found a qualitative correlation (94.3% agreement), we did not observe a quantitative correlation between IFN-γ and anti-S1 IgG levels (rho = 0.14) [35–37].

Unlike Alexander Krüttgen and colleagues, who propose a lower cut-off in order to increase the sensitivity of another IGRA assay (QuantiFERON SARS-CoV-2 assay, Qiagen, Düsseldorf, Germany), in our study population of vaccinated healthcare workers this commercial cut-off was adequate to find high sensitivity values [35]. Perhaps other types of populations, such as immunocompromised patients, could benefit from lower cut-offs, but we did not make this assessment.

Using stimulated whole blood from a subset of participants, we confirmed the specific T-cell response to SARS-CoV-2 by dosing elevated levels of the IL-6, IL-10 and TNF-α (Fig 3). The Cytokine Release Syndrome is indeed described in the response to COVID-19 [38, 39]. High levels of IL-6, IL-10 and TNF-α have been associated with more severe forms of the disease [38, 40–44]. We have to note that we assessed interleukins from plasma stimulated through the IGRA tube. Thus, these interleukin levels cannot be compared to those described by other authors for direct dosing from COVID-19 patients serum (IL-6: 19.55 pg/mL mean; IL-10: 3.66 pg/mL mean; TNF-α: 1.11 pg/mL mean) or vaccinated participants serum (IL-6: 25.94 pg/mL mean; IL-10: 11.08 pg/mL mean) [45–47]. However, interestingly, we found that IL-6, which has been described as the strongest interleukin predicting disease severity, was the most expressed in our study [38, 40–44]. Because IL-6 and IL-10 participate in the differentiation of activated B lymphocytes towards plasma cells, we looked for a correlation between their levels and those of IgG and no correlation was found. Future studies in a larger number of patients and vaccine recipients will be needed to establish possible mechanisms.

Through participant follow-up, we found that among 43% who had COVID-19, none had severe illness requiring hospitalization. Furthermore, the first positive cases were only 12 months after the 2nd dose inoculation (Fig 4A). Interestingly, the periods of increased infection in the healthcare workers enrolled corresponded to national infection peaks, namely January and May 2022 (Fig 4B and 4C). These two periods are linked to the switch from the Delta variant to Omicron BA.1/BA.2 and from the latter to the Omicron BA.5 strain, which may explain the COVID-19 pronounced increase in both the community and healthcare workers (Fig 4A). Only 2 of our participants were IGRA-negative, so it is not possible to establish a qualitative relationship between the IGRA result and future COVID-19. A quantitative relationship through IGRA levels was not found either. Further studies will be necessary.

Although we believe that our study adds knowledge in the fields of vaccine immune response and COVID-19 diagnosis, it has several limitations. Perhaps because the number of women (75%) is much higher than men among the professionals at our hospital, the amount of women who volunteered was also much higher than men. Unfortunately, a control group to access the specificity of IGRA was not included. The test was not evaluated for different vaccine types, nor in patients, namely the immunosuppressed ones. Cellular response was not measured over time to establish the kinetics of IFN-γ production and to look for possible associations in the course of the immune response. Cellular response was not correlated with the neutralizing antibodies.

In recent years, IGRA-TB has been an important tool for the diagnosis of latent tuberculosis and treatment management, particularly in those with a high degree of exposure to TB and in immunosuppressed patients or those starting immunosuppressive therapies. Future studies are needed to understand if IGRA-SARS-CoV-2 can be a powerful diagnostic tool to predict future COVID-19 infection and especially severe disease, both in immunocompetent and immunosuppressed patients, even in the absence of antibodies. If so, this simple and easy-to-perform test, which can be implemented in clinical laboratories, could be very useful for guiding vaccination policies, such as determining intervals between vaccine boosters and priority target populations.

## Conclusions

Through an IGRA, we found that 6 months after inoculation of the 2nd dose of BNT162b2 vaccine, more than 94% of healthcare workers have a positive cellular immune response to SARS-CoV-2. A qualitative but not quantitative correlation was found between the levels of IFN-γ and IgG produced against the virus. Elevated levels of IL-6, IL-10 and TNF-α, confirm the memory cell immune response via T cells. During a 20-month follow-up, among the 43% of participants who had COVID-19, none had severe disease. Moreover, positive cases started to appear only 1 year after the 2nd shot was taken. Further studies are needed to fully understand whether this simple test to assess immune cell response may be useful to, for example, determine the most efficient intervals between different doses or boosters of the vaccine and identify the highest priority target populations.

## Supporting information

S1 File Study population characterization. The S1 File shows the characterisation of the study population based on analytical parameters such as hemogram, as well as hepatic and renal biomarkers.
(XLSX)

S2 File Evolution of anti-S1 IgG levels after vaccine inoculation. The S2 File shows the anti-S1 IgG levels assessed at the first and sixth month after the administration of the 2nd dose of the COVID-19 vaccine.
(XLSX)

S3 File Correlation between IgG and IGRA levels. The S3 File shows the IgG and IGRA values determined 6 months after the inoculation of the 2nd dose of the COVID-19 vaccine. The correlation was performed among the 116 samples that were positive for IGRA determination.
(XLSX)

S4 File Interleukin levels and the corresponding IgG and IGRA levels. The S4 File shows the interleukin values measured in 18 healthcare workers, 6 months after the 2nd dose of the COVID-19 vaccine. The corresponding IgG and IGRA levels are also shown.
(XLSX)

S5 File Monthly new cases of COVID-19 in Portugal and in CHUSJ. The S5 File shows the amount of COVID-19 cases both nationally and at our hospital between December 2020 and September 2022. Regarding hospital cases, they are reported as the total number of positive cases in each month. As for the country-wide cases, they are reported as the total number of positive cases per month and the average number of positive cases per month. The graph corresponding to the national data is presented using the average (Fig 4B).
(XLSX)

S6 File Correlation of sex and age with IGRA levels. The S6 File shows the IGRA values measured 6 months after the 2nd dose of the COVID-19 vaccine and the corresponding demographic data such as gender and age.
(XLSX)
